# Characterization of a new wheat food allergen

**DOI:** 10.1186/2045-7022-1-S1-P86

**Published:** 2011-08-12

**Authors:** Sandra Pahr, Claudia Constantin, Nikos Papadopoulus, Mika Makela, Christof Ebner, Adriano Mari, Irene Mittermann, Susanne Vrtala, Rudolf Valenta

**Affiliations:** 1 Christian Doppler Laboratory for the development of allergen chips, Department of Pathophysiology and Allergy Research, Medical University of Vienna, Vienna, Austria; 2 Department of Pathophysiology and Allergy Research, Medical University of Vienna, Vienna, Austria; 3 Allergy Department, 2nd Pediatric Clinic, University of Athens, Athens, Greece; 4 Skin and Allergy Hospital, Helsinki University Central Hospital, Helsinki, Finland; 5 Ambulatory for Allergy and Clinical Immunology, Vienna, Austria; 6 Center for Clinical and Experimental Allergology, IDI-IRCCS, Rome, Italy; 7 Christian Doppler Laboratory for Allergy Research, Department of Pathophysiology and Allergy Research, Medical University of Vienna, Vienna, Austria

## Background

Allergens from wheat (Triticum aestivum) can cause three distinct IgE-mediated allergies: respiratory allergy by inhalation of wheat flour, wheat pollen allergy and wheat food allergy. Wheat and wheat products are a main component in human diet, but currently avoidance is the only therapy for wheat food allergic patients. Diagnosis based on wheat flour extracts does not discriminate between patients suffering from respiratory allergy or wheat food allergy. Aim of this study was the isolation, identification and characterization of new allergens recognized by patients suffering from wheat food allergy to establish methods and diagnostic tests to specifically identify wheat food allergic patients.

## Methods

cDNA clones coding for wheat allergens could be isolated from a Triticum aestivum expression cDNA library by screening with serum IgE antibodies from wheat food allergic patients. The cDNA coding for a novel wheat food allergen, alpha-purothionin, could be identified. Recombinant alpha-purothionin was expressed, purified and characterized regarding molecular, structural and immunological properties. Allergen specific rabbit antibodies were used to screen other cereal extracts for homologues proteins. Inhibition assays were performed with serum from wheat food allergic patients.

## Results

We could isolate alpha-purothionin from a cDNA expression library and identified the C-terminal acidic extension domain as IgE-epitope containing portion. The novel wheat food allergen was expressed as recombinant protein in E.coli and the IgE-binding of alpha-purothionin was confirmed in IgE-dot blot experiments. Homologue proteins to alpha purothionin could be detected by allergen specific rabbit antibodies in many other cereal extracts, accordingly cross-reactivity can be observed.

## Conclusion

Recombinant alpha-purothionin may be useful for the diagnosis and possibly immunotherapy of IgE-mediated wheat food allergy.

Supported by Phadia, Uppsala, Sweden and the Christian Doppler Research Association, Austria.

